# Temperature Dependence on Microstructure, Crystallization Orientation, and Piezoelectric Properties of ZnO Films

**DOI:** 10.3390/s25010242

**Published:** 2025-01-03

**Authors:** Ke Deng, Zhonghao Liu, Hulin Liu, Yanxiang Chen, Shang Li, Shuren Guo, Boyu Xiu, Xuanpu Dong, Huatang Cao

**Affiliations:** 1 Zhuzhou Hanjie Aviation Science & Technology Co., Ltd., Zhuzhou 412002, China; 13317335666@163.com (K.D.); yxchen@vip.163.com (Y.C.); 2 State Key Laboratory of Materials Processing and Die & Mould Technology, School of Materials Science and Engineering, Huazhong University of Science and Technology, Wuhan 430074, China; m202471128@hust.edu.cn (H.L.); shangli@hust.edu.cn (S.L.); gsr@hust.edu.cn (S.G.); dongxp@hust.edu.cn (X.D.); 3 Shanghai Research Institute of Materials Co., Ltd., Shanghai 200437, China; xiuboyu@163.com

**Keywords:** ZnO films, annealing treatment, microstructure, piezoelectric, sensors

## Abstract

This study has investigated the effects of different annealing temperatures on the microstructure, chemical composition, phase structure, and piezoelectric properties of ZnO films. The analysis focuses on how annealing temperature influences the oxygen content and the preferred c-axis (002) orientation of the films. It was found that annealing significantly increases the grain size and optimizes the columnar crystal structure, though excessive high-temperature annealing leads to structural degradation. This behavior is likely related to changes in oxygen content at different annealing temperatures. High resolution transmission electron microscopy (HR-TEM) reveals that the films exhibit high-resolution lattice stripes, confirming their high crystallinity. Although the films exhibit growth in multiple orientations, the c-axis (002) orientation remains the predominant crystallographic growth. Further piezoelectric property analysis demonstrates that the ZnO films annealed at 400 °C exhibit enhanced piezoelectric performance and stable linear piezoelectric behavior. These findings offer valuable support for optimizing the piezoelectric properties of ZnO films and their applications in piezoelectric sensors.

## 1. Introduction

The piezoelectric properties of ZnO films arise from their non-centrosymmetric, tetrahedrally coordinated hexagonal wurtzite structure [1,2]. This distinctive structure endows ZnO with exceptional piezoelectric characteristics, making it a focal point of research in applications such as sensors [3], micro-electromechanical systems (MEMS) [4], and ultrasonic transducers [5]. Notably, these piezoelectric properties are highly sensitive to the structural features and crystallographic orientation of the film [6]. The growth of the film along the c-axis (002) orientation is of paramount importance, as it ensures the optimal electromechanical coupling efficiency, which is a critical factor for achieving exceptional piezoelectric performance [7]. These structural attributes are highly sensitive to the fabrication conditions, including the selection of the preparation process and annealing heat treatment, which significantly influences the crystallographic orientation and microstructure of the films [8,9,10].

Magnetron sputtering is a technique widely employed for the deposition of ZnO films. It enables the production of uniform, dense, and high-quality films at room temperature [11,12]. The structure and crystal orientation of ZnO films can be significantly optimized by adjusting the sputtering process, including sputtering power, substrate temperature, post-deposition treatments, etc. [13,14,15,16]. Among the various factors affecting the structure and piezoelectric properties of ZnO films, temperature is a significant contributor. During the deposition process, the substrate temperature significantly affects the mobility, nucleation, and growth mechanisms of depositing atoms, which collectively determine the film’s crystallinity and orientation [14,17]. However, excessively high substrate temperatures can introduce significant thermal stress, which is detrimental to the bonding between the film and the substrate [18]. Post-deposition temperature treatment effectively alleviates residual stress within the films and significantly enhances the piezoelectric properties of ZnO films by improving crystal quality, optimizing crystal orientation, and regulating oxygen content. For instance, G.P. Daniel et al. [19] fabricated ZnO films using radio frequency magnetron sputtering at room temperature and subsequently annealed them in air for 1 h to regulate the grain size and crystal orientation of the films. Liu et al. [20] demonstrated that ZnO films exhibit strong c-axis orientation and increased resistivity in oxygen-rich environments, primarily due to the formation of acceptor-type defects, mainly Zn vacancies (V_Zn_). These defects become mobile at temperatures exceeding 250 °C and can be effectively eliminated through post-deposition annealing in a vacuum condition at 300 °C, resulting in a significant enhancement in the films’ optoelectronic properties. However, the intrinsic relationship between the oxygen content, c-axis orientation, and piezoelectric properties of ZnO films prepared by magnetron sputtering and subsequent annealing remains unexplored. Additionally, the underlying mechanisms of the influence of different temperatures on films require further investigation. This work aims to investigate the mechanisms by which annealing at different temperatures influences ZnO films prepared via magnetron sputtering, and to analyze the microstructural evolution of the films following annealing treatment, thereby elucidating the mechanisms underlying the enhancement of their piezoelectric properties.

In this work, ZnO films were fabricated using radio frequency (RF) magnetron sputtering, and the films were subsequently annealed at varying temperatures to examine the evolution of their microstructure, oxygen content, and crystallographic orientation. The study aims to correlate these structural changes with the piezoelectric properties of the films, ultimately identifying the optimal annealing conditions for enhancing their performance.

## 2. Materials and Methods

The ZnO film was fabricated using radio frequency magnetron sputtering and deposited onto the surface of a Si substrate. Prior to being placed in the sputtering vacuum chamber, the substrate surface was cleaned and was subjected to ultrasonic cleaning in an alcohol solution for 30 min, before then being dried in a constant-temperature drying oven at 60 °C for 30 min. Additionally, Ar plasma cleaning was conducted inside the sputtering vacuum chamber with the following parameters to remove impurities from the substrate surface: a bias voltage of −300 V, a duty cycle of 72%, and a period of 10 min. The ZnO film was deposited using a ZnO target with 99.99% purity and a diameter of 4 inches. The deposition parameters were as follows: a maintained base pressure of 1.0 × 10^−4^ Pa, a target-substrate distance of 60 mm, a sputtering power of 50 W, a working pressure of 1.0 Pa, a deposition atmosphere of high-purity Ar (99.999%), and a deposition time of 10 h. The deposited films were subsequently annealed in air at temperatures of 100 °C, 200 °C, 300 °C, 400 °C, 500 °C, and 600 °C, with an annealing duration of 1 h. A ZnO film prepared under identical deposition conditions without annealing was used as a reference for comparison.

The surface morphology and cross-sectional microstructure of the ZnO film deposited on the Si substrate were analyzed using scanning electron microscopy (SEM, JSM-IT800, JEOL Ltd. Manufacturing company, Akishima, Tokyo, Japan). The chemical composition of the film was determined using energy-dispersive X-ray spectrometry (EDS) integrated with the JSM-IT800. All measurements were conducted at an acceleration voltage of 10 kV. The phase composition of the ZnO film was analyzed using grazing incidence X-ray diffraction (GI-XRD) with a Rigaku SmartLab 9KW system (Rigaku Corporation, Tokyo, Japan). Measurements were performed at a grazing incidence angle of 1° over a 2θ scanning range of 10–80°. The phase and crystal structure of the ZnO film were further characterized using high-resolution transmission electron microscopy (HR-TEM, Talos F200X, Thermo Fisher Scientific, Waltham, MA, USA). The TEM samples of the unannealed ZnO film were prepared using sectioning and thinning techniques with focused ion beam (FIB, Helios 5 Dual Beam, Thermo Fisher Scientific, Waltham, MA, USA) microscopes. The piezoelectric properties of ZnO films were characterized using a multifunctional atomic force microscope (AFM, Jupiter XR, Oxford Instruments, Abingdon, UK) operating in piezoelectric force microscopy (PFM) mode. PFM measurements were conducted with a 1 V drive voltage, a Pt/Ir-coated conductive tip, a force constant of 3 N/m, and a resonant frequency of 300 kHz. Additionally, a bias voltage ranging from −8 V to 8 V was applied to a single piezoelectric domain to assess changes in amplitude and phase. To ensure the reliability and reproducibility of the results, all the aforementioned tests were performed on three parallel samples fabricated under identical conditions.

## 3. Results and Discussions

### 3.1. Structural and Composition of ZnO Films

**Figure 1:**
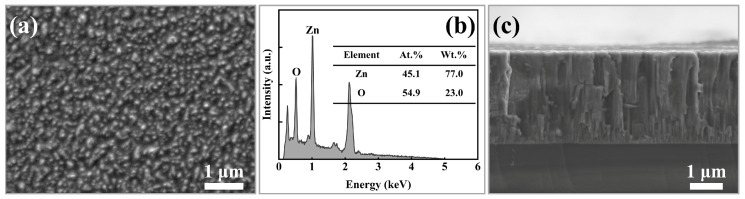
(**a**) Surface morphology, (**b**) chemical composition, and (**c**) fractured cross-section of unannealed ZnO films.

Figure 1 illustrates the surface morphology, chemical composition, and cross-sectional structure of the unannealed ZnO film. The film exhibits typical cauliflower-like structural characteristics of sputtered films, including a dense microstructure free of pores and cracks, with nanoscale grain sizes, as shown in Figure 1a. These structural features are consistent with those reported for magnetron-sputtered ZnO films in previous studies. The smaller grain size is beneficial for maintaining smaller piezoelectric domains, which affects domain wall flipping and enables a rapid piezoelectric response [21]. ZnO films possess a non-centrosymmetric structure with the tetrahedral coordination of Zn and O atoms, which serve as the primary origin of their piezoelectric properties. The optimal Zn/O atomic ratio is 1:1, ensuring a stable crystal structure and optimal electrical properties [22]. Figure 1b presents the chemical composition of the unannealed ZnO film, where the atomic percentages of Zn and O are 45.1 at.% and 54.9 at.%, respectively, corresponding to a Zn/O ratio of 0.82. This ratio is close to the ideal stoichiometric value of 1:1. Figure 1c illustrates the cross-sectional structure of the ZnO film, revealing a well-defined and dense columnar crystal structure with a film thickness of 2.8 μm. In piezoelectric films, such a columnar structure facilitates a shorter electric field transmission path and ensures uniform mechanical coupling, enabling the film to achieve rapid responses to external forces [23].

**Figure 2:**
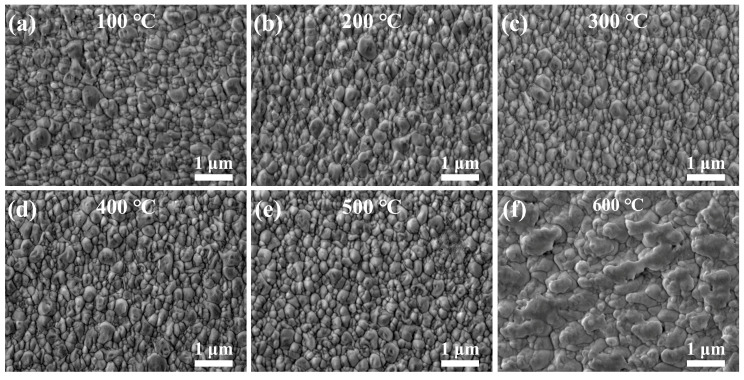
Surface morphology of ZnO films annealed at different temperatures: (**a**) 100 °C, (**b**) 200 °C, (**c**) 300 °C, (**d**) 400 °C, (**e**) 500 °C, and (**f**) 600 °C.

To investigate the effect of annealing temperature on the surface morphology of ZnO films, Figure 2 illustrates the surface morphology of the films subjected to different annealing temperatures. The annealed films exhibit a more pronounced cauliflower structure, which remains dense and free from cracks and pores. However, the grain size increases significantly compared to the unannealed films. The grain size of the films annealed at temperatures ranging from 100 °C to 500 °C shows little sensitivity to temperature variations, as depicted in Figure 2a–e. In contrast, annealed at 600 °C, the grain size increases further, and the cauliflower structure becomes less distinct. Grain coalescence due to melting was evident, resulting in a highly interconnected microstructure, as shown in Figure 2f. This phenomenon reduces the number of grain boundaries, disrupts microstructural homogeneity, and alters stress distribution, thereby adversely impacting the piezoelectric properties of ZnO films [15].

**Figure 3:**
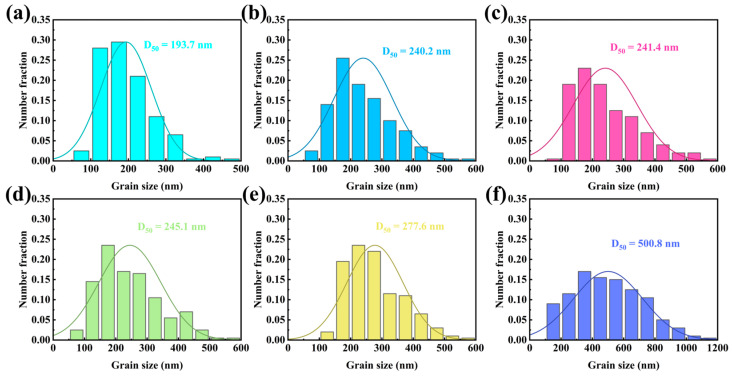
Grain size of ZnO films annealed at different temperatures: (**a**) 100 °C, (**b**) 200 °C, (**c**) 300 °C, (**d**) 400 °C, (**e**) 500 °C, and (**f**) 600 °C.

To intuitively evaluate the effect of annealing treatment on the grain growth of ZnO thin films, the grain size was measured and subjected to statistical analysis. Figure 3a–f presents the grain sizes of ZnO thin films annealed at temperatures ranging from 100 °C to 600 °C. The results indicate that the average grain size increases systematically with an annealing temperature, from 193.7 nm at 100 °C to 500.8 nm at 600 °C. The grain size distribution for all samples exhibits a normal distribution. This increase in grain size is primarily attributed to the annealing process, which promotes atomic diffusion, stress relaxation, and defect reduction.

**Figure 4:**
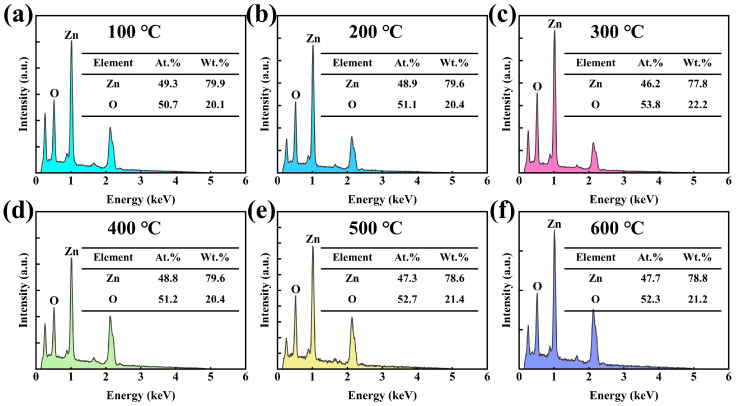
Chemical compositions of ZnO films annealed at different temperatures: (**a**) 100 °C, (**b**) 200 °C, (**c**) 300 °C, (**d**) 400 °C, (**e**) 500 °C, and (**f**) 600 °C.

Figure 4 presents the chemical composition of the film annealed at different temperatures, with particular emphasis on changes in oxygen content to assess the influence of annealing temperature. The oxygen content of the annealed film shows a noticeable reduction compared to that of the unannealed film (as shown in Figure 1b). This reduction is likely due to the annealing process, which facilitates the excitation of oxygen atoms within the film, causing them to detach from the lattice and form oxygen vacancies, thereby decreasing the overall oxygen content [24]. However, at lower annealing temperatures (100–300 °C), the oxygen content gradually increases with a rising annealing temperature, as shown in Figure 4a–c. This behavior may be attributed to enhanced oxygen adsorption on the film during the air annealing process, leading to a corresponding increase in oxygen content [25]. As the annealing temperature increases, the oxygen content of the film decreases. As shown in Figure 4d–f, higher temperatures lead to the increased excitation of oxygen atoms within the film lattice, resulting in the formation of more oxygen vacancies [24]. This effect outweighs the oxygen adsorption from the air, leading to a reduction in the overall oxygen content.

**Figure 5:**
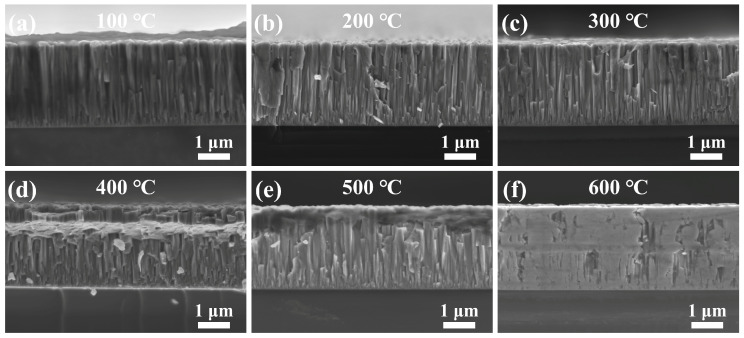
Fractured cross-sections of ZnO films annealed at different temperatures: (**a**) 100 °C, (**b**) 200 °C, (**c**) 300 °C, (**d**) 400 °C, (**e**) 500 °C, and (**f**) 600 °C.

To further investigate the impact of different annealing temperatures on the columnar crystal structure of the film and achieve a well-defined and dense columnar arrangement, Figure 5 presents the fractured cross-sections of the film annealed at different temperatures. The film thickness remains at approximately 2.8 μm, indicating that the annealing treatment has minimal effects on the alteration of the film thickness. After annealing at temperatures ranging from 100 °C to 400 °C, the fractured cross-sectional structure of the film shows no significant alterations, maintaining a clear and dense columnar crystal structure. This columnar structure is more defined and compact than that of the unannealed ZnO film, as shown in Figure 5a–d. However, with further increases in annealing temperature, the fractured cross-sectional structure gradually exhibits signs of grain melting near the surface, as shown in Figure 5e. Notably, at 600 °C, the columnar structure is lost due to the melting and coalescence of the grains, as shown in Figure 5f. The change in the columnar structure may result from the growth and rearrangement of grains at lower annealing temperatures (such as 100 °C to 400 °C), which reduces grain boundary defects. This rearrangement typically enhances the density and structural uniformity of the material. However, at higher temperatures, the heat causes the grains to melt, leading to grain coalescence and the gradual disappearance of the columnar crystal structure [26].

### 3.2. Crystal Orientation of ZnO Films

**Figure 6:**
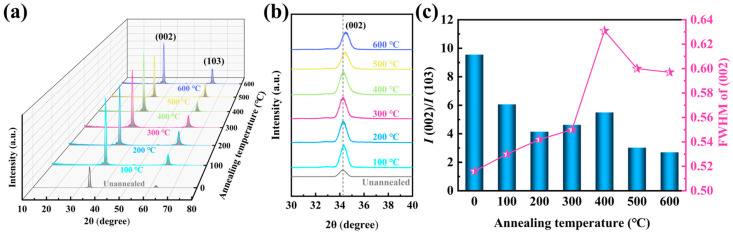
GI-XRD patterns of ZnO films annealed at different temperatures, shown for the scanning ranges of (**a**) 10–80° and (**b**) 30–40°, along with the (**c**) corresponding orientation intensity ratio and FWHM of the (002) crystal plane.

To investigate the influence of different annealing temperatures on the physical phase of ZnO films, the GI-XRD patterns of the films are presented in Figure 6a. All films exhibit distinct sharp peaks near 2θ = 34° and a smaller peak near 2θ = 62°, corresponding to the (002) and (103) orientations of ZnO, respectively, as identified by comparison with JCPDS standard card no. 00-036-1451. The unannealed film exhibits a strong c-axis (002) orientation and a negligible (103) orientation. Annealed at relatively low temperatures (100–400 °C), the (002) orientation of the ZnO film is significantly strengthened. However, as the annealing temperature exceeds 400 °C, an orientation transition occurs, with a gradual decrease in the (002) orientation and a corresponding increase in the (103) orientation. This phenomenon can be attributed to the hexagonal wurtzite structure of the ZnO film, in which the (002) crystal plane exhibits the lowest surface free energy along the c-axis direction, making it a thermodynamically stable orientation. During the annealing process, grain rearrangement occurs as the system’s free energy is minimized, which preferentially enhances the c-axis orientation. However, as the annealing temperature increases beyond 400 °C, the concentration of oxygen vacancies increases, resulting in altered defect density and stress distribution within the ZnO lattice, thereby inhibiting the growth of the c-axis orientation [27,28,29]. Figure 6b illustrates the shift in the (002) peak near 2θ = 34° with varying annealing temperatures. It can be observed that the (002) orientation peak of the film gradually shifts to a higher angle when annealed at temperatures above 400 °C, further supporting the notion that the concentration of oxygen vacancies increases with higher annealing temperatures. During the annealing process, the formation of oxygen vacancies alters the ZnO lattice structure, causing a slight contraction of the lattice, which results in the XRD peak shifting towards higher angles [24]. Figure 6c presents the ratio of the (002) orientation intensity to the (103) orientation intensity I(002)/I(103) of the film annealed at different temperatures, along with the full width at the half maximum (FWHM) of the (002) orientation peak. As the annealing temperature increases, the proportion of the (002) orientation in the film gradually decreases, although the (002) intensity increases significantly. The observed trend in the FWHM is primarily attributed to the formation of oxygen vacancies during the annealing process, as well as the influence of oxygen adsorption on the crystallization quality.

**Figure 7:**
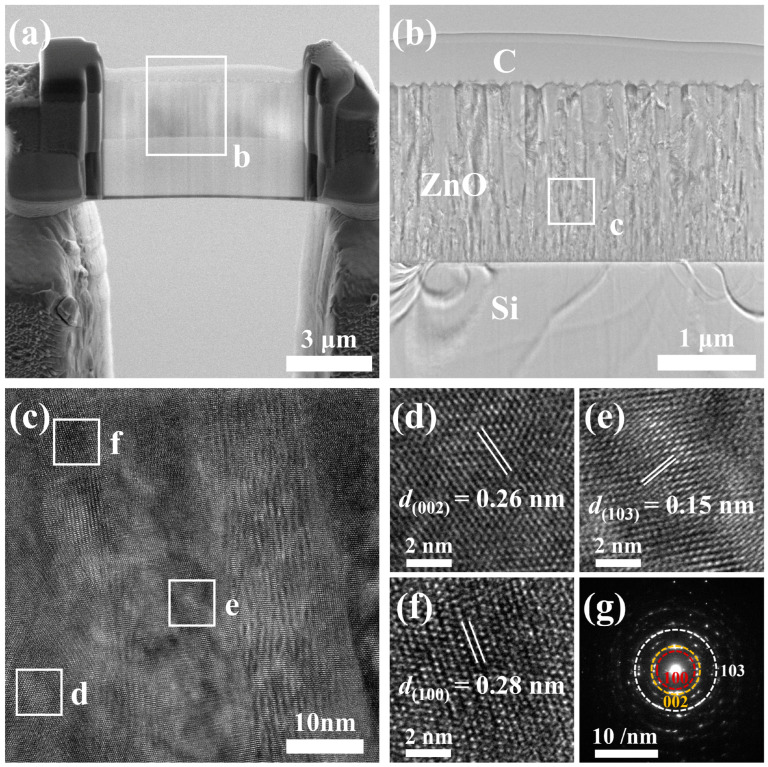
(**a**) FIB-SEM image of the cross-section of the unannealed ZnO film prepared by FIB cutting, (**b**) close-up view of the indicated sliced cross-section, (**c**) HR-TEM image of the film, (**d**–**f**) IFFT spectra corresponding to different crystal plane arrangements, and (**g**) diffraction pattern of the film.

To further investigate the microstructure of the magnetron-sputtered ZnO film, TEM thin sections were prepared via the FIB cutting of the unannealed film. Figure 7a presents an overview of the cross-sectional thin section. The sample thickness was precisely controlled to achieve an ultra-thin electron transparent specimen (on the order of a few tens of nanometers), meeting the requirements for TEM analysis. A magnified view of the cross-section is shown in Figure 7b, revealing a clear and dense columnar crystal structure growing from the surface of the silicon substrate. The top layer of the film is the protective carbon layer deposited during the FIB sample preparation. The HR-TEM image of the ZnO film is presented in Figure 7c, corresponding to region c in Figure 7b. The atomic structure of the ZnO film is characterized by well-resolved lattice fringes. The uniformity of these fringes confirms the high crystallinity of the film, while variations in crystal plane arrangements indicate the presence of multiple crystal orientations. Figure 7d–f display the inverse fast Fourier transform (IFFT) spectra corresponding to different crystal plane arrangements, labeled as regions d, e, and f in Figure 7c, respectively. The IFFT spectra of the ZnO film reveal well-defined and periodic lattice fringes in different regions, with interplanar spacings of 0.26 nm, 0.15 nm, and 0.28 nm, corresponding to the (002), (103), and (100) crystal planes, respectively. This confirms the growth orientation of the film along the (002) and (103) directions, which is consistent with the results obtained from GI-XRD analysis. Figure 7g presents the diffraction pattern of the ZnO film, exhibiting multiple concentric diffraction rings, a characteristic feature of a polycrystalline structure. These rings correspond to the (002), (103), and (100) crystal planes of ZnO. The intensity distribution along the rings indicates that the grains in the film are predominantly crystal-oriented along the c-axis (002) direction.

### 3.3. Piezoelectric Properties of ZnO Films

**Figure 8:**
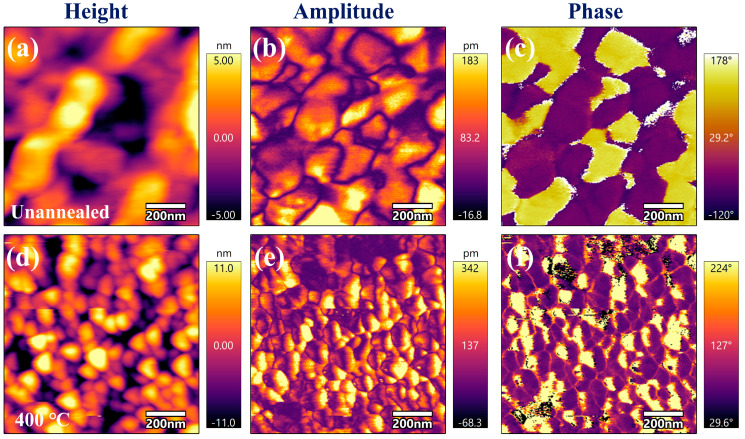
PFM analysis of ZnO films: (**a**) height, (**b**) amplitude, and (**c**) phase images of unannealed films, as well as the (**d**) height, (**e**) amplitude, and (**f**) phase images of films annealed at 400 °C.

To evaluate the enhancement of the piezoelectric properties of ZnO films through annealing, PFM piezoelectric analysis was conducted on both the unannealed film and the film annealed at 400 °C. The film annealed at 400 °C exhibits the strongest c-axis (002) orientation, as shown in Figure 6a. The height, amplitude, and phase images of the unannealed and 400 °C annealed films are presented in Figure 8a–c and Figure 8d–f, respectively. All the films exhibit nanoscale roughness, with the roughness of the annealed film being higher, which is attributed to grain growth. The amplitude and phase images of the ZnO films were obtained by applying the same excitation voltage in PFM mode. In the amplitude images, different color contrasts correspond to varying amplitudes and their associated piezoelectric responses [30]. The phase image reveals distinct bright and dark regions, indicating different polarization directions and piezoelectric phase distributions [31]. Comparing the amplitude images of the film before and after annealing (Figure 8b,e), the piezoelectric amplitude of the film after annealing at 400 °C significantly increases (from a central amplitude of 83.2 pm to 137 pm), confirming that the annealing treatment notably enhances the c-axis orientation and piezoelectric response of the ZnO film. The phase change shown in Figure 8c,f is a prerequisite for the film to exhibit piezoelectric behavior [32].

**Figure 9:**
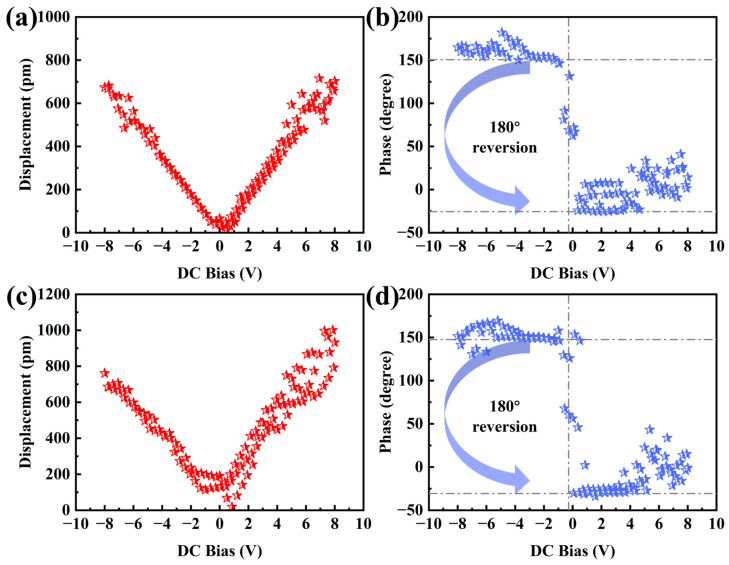
(**a**) Displacement–voltage relationship and (**b**) phase–voltage relationship of the unannealed ZnO film; (**c**) displacement–voltage relationship and (**d**) phase–voltage relationship of the ZnO film annealed at 400 °C.

Figure 9 illustrates the variation in the piezoelectric amplitude and phase of a single piezoelectric domain in the unannealed and 400 °C annealed films under a bias ranging from −8 V to 8 V. The amplitude of the ZnO film exhibits a linear relationship with the applied bias, which enhances the electromechanical response and conversion efficiency of the piezoelectric film [33]. As shown in Figure 9a,c, the maximum amplitude of the annealed film, under the same applied bias, favorably exceeds that of the unannealed film, highlighting the enhancement in piezoelectric response thanks to annealing. This indicates that annealing improves the piezoelectric response of the film while preserving its linear piezoelectric behavior. Figure 9b,d presents the piezoelectric amplitude variation curves of the unannealed and 400 °C annealed films, respectively, under the same applied bias. As the bias transitions from negative to positive, the piezoelectric polarity undergoes a reversal. This polarity change corresponds to an approximately 180° phase reversal, occurring at the instant the bias transitions from negative to positive. Such behavior is a critical prerequisite for the film to exhibit a linear piezoelectric response [34].

## 4. Conclusions

In this study, ZnO piezoelectric films were fabricated using radio frequency magnetron sputtering, followed by annealing at different temperatures. The effects of annealing temperature on the films’ surface morphology, chemical composition, fractured cross-sectional structure, phase structure, and piezoelectric properties were systematically investigated. The underlying enhancement mechanisms of piezoelectric properties through annealing were also thoroughly discussed. The main findings are summarized as follows:

1. Annealing treatment can significantly enhance the grain size of ZnO films and improve the density and structural characteristics of the columnar crystals. However, when annealed at higher temperatures (600 °C), the film grains undergo melting, leading to noticeable grain aggregation and the loss of the columnar structure. Additionally, annealing at various temperatures influences the oxygen content and helps control the formation of oxygen vacancy defects.
2. The c-axis (002) orientation of the film is significantly enhanced by annealing treatment, but it begins to decrease after annealing at 400 °C. As the annealing temperature increases, the peak corresponding to the (002) crystal plane shifts to a higher angle, indicating the effect of oxygen vacancy formation on the film’s physical phase. HR-TEM analysis reveals that the film exhibits multiple growth orientations, with the c-axis (002) orientation being the most dominant.
3. The annealing treatment significantly enhances the piezoelectric response of the film, as evidenced by a higher piezoelectric amplitude under the same voltage stimulation. This improvement is accompanied by a stable linear piezoelectric response and rapid phase polarization switching. The strong c-axis (002) orientation is confirmed to contribute to the enhancement of the piezoelectric properties.

## Data Availability

Data are contained within the article.
